# Force‐field parametrization based on radial and energy distribution functions

**DOI:** 10.1002/jcc.26035

**Published:** 2019-07-25

**Authors:** Shuntaro Chiba, Yasushi Okuno, Teruki Honma, Mitsunori Ikeguchi

**Affiliations:** ^1^ RIKEN Medical Sciences Innovation Hub Program, 1‐7‐22, Suehiro‐cho, Tsurumi‐ku Yokohama 230‐0045 Japan; ^2^ RIKEN Cluster for Science and Technology Hub, 6‐3‐5, Minatojima‐minamimachi, Chuo‐ku, Kobe Hyogo 650‐0047 Japan; ^3^ Graduate School of Medicine Kyoto University Shogoin‐Kawaharacho, Sakyo‐ku Kyoto 606‐8507 Japan; ^4^ Graduate School of Medical Life Science Yokohama City University, 1‐7‐29, Suehiro‐cho, Tsurumi‐ku Yokohama 230‐0045 Japan

**Keywords:** force field parametrization, coarse graining, iterative Boltzmann inversion, Newton inversion, energy representation

## Abstract

We propose a novel force‐field‐parametrization procedure that fits the parameters of potential functions in a manner that the pair distribution function (DF) of molecules derived from candidate parameters can reproduce the given target DF. Conventionally, approaches to minimize the difference between the candidate and target DFs employ radial DFs (RDF). RDF itself has been reported to be insufficient for uniquely identifying the parameters of a molecule. To overcome the weakness, we introduce energy DF (EDF) as a target DF, which describes the distribution of the pairwise energy of molecules. We found that the EDF responds more sensitively to a small perturbation in the pairwise potential parameters and provides better fitting accuracy compared to that of RDF. These findings provide valuable insights into a wide range of coarse graining methods, which determine parameters using information obtained from a higher‐level calculation than that of the developed force field. © 2019 The Authors. *Journal of Computational Chemistry* published by Wiley Periodicals, Inc.

## Graphical abstract

Radial distribution function (RDF) and energy distribution function (EDF) are used to parameterize a target molecule's force field parameters based on the fundamental Henderson theorem, which proves the one‐to‐one correspondence between their pairwise potential and the resulting distribution function. The study shows that the EDF responds more sensitively to a small perturbation added to the pairwise potential parameters and the fitting accuracy was better compared to that obtained with the RDF‐based fitting.

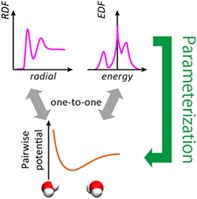

## Introduction

Molecular dynamics (MD) simulation has become a powerful and easy‐to‐use tool to understand the molecular processes.^1^ It simulates the temporal development of molecules according to the equation of motion, using forces derived from energy functions with their parameters for molecules, referred to as a force field (FF). Parametrization is conducted so that the parameters can reproduce the target properties of molecules of interest. The target properties employed depend on the parametrization policy of a certain FF. Experimental and/or computed information is used for the target properties. The experimental information used includes crystallography, vibrational spectroscopy, NMR, enthalpy of vaporization, density of liquid phase, and partitioning coefficient of different liquids.^2^, ^3^ Computed information is derived from higher‐level calculation than that of the developed FF, such as by quantum mechanical calculation;^2^, ^3^, ^4^ for some coarse‐grained FFs, information derived from a fine‐grained FF is used.^5^

Parametrization of FFs is considered essential, and various methods and variations of parameters are still being developed actively. For example, the water molecule is the most active target of parameterization.^6^ A study regarding the SPC/E water model^7^ reported that a 1% increase in the length of the OH bond led to accurate reproducibility of the translational and rotational diffusion of pure water and proteins. This newly developed model was named SPC/E_b_.^8^ In the TIP4P/2005 water model,^9^ an increase in the dispersion term followed by adjusting the repulsive term improved the structure reproducibility of disordered proteins,^10^ which was named as TIP4P‐D and was revised recently.^11^

In this study, we propose an approach that can automatically determine the parameters of a molecule. A molecule of interest is described with energy functions of bonded and nonbonded parameters in an additive fashion as done in common FFs such as AMBER FFs.^12^, ^13^ The method uses pair distribution functions (DFs) of the target molecule represented in the radial axis as the radial distribution function (RDF), and/or in the energy axis as the energy distribution function (EDF), which are calculated by other computational methods. Using this approach, we can automatically determine the parameters of a molecule using the DFs obtained from a higher‐level calculation than that of the developed FF. This approach fits the candidates' parameters of the molecule so that the derived RDF and/or EDF can reproduce the target RDF and/or EDF to the greatest extent. This method is based on the fundamental Henderson theorem,^14^ which proves the one‐to‐one correspondence between the pairwise interaction potential of molecules and the corresponding pair DF. So far, the RDF has been used as the target property for parameterization of additive FFs and its numerous applications have been reported. In particular, it is used to determine coarse‐grained FF parameters from the RDF derived with a fine‐grained FF, using the iterative Boltzmann inversion (IBI),^15^ the inverse Monte Carlo approaches,^16^ or the Newton inversion.^17^ However, some studies have reported that very similar RDFs gave different interaction potentials.^18^, ^19^, ^20^ To confine the space to be searched for candidate parameters that give similar RDFs, the use of additional information is proposed, for example, the pressure of the system^18^ and RDFs derived from multiple thermodynamic states.^19^

We hypothesized that the EDF, compared to the RDF, would respond more sensitively to changes in interaction parameters. We then introduced the EDF as a target property, expecting that the fitting would be more successful. Moreover, this EDF‐based approach can remove the dependency of fitting results on the cutoff definition that the RDF‐based approach has,^21^ because pairwise interaction energy falls within a limited range. Note that the Henderson theorem holds true for various DFs,^14^ such as the EDF,^22^ in addition to the RDF.

To test the validity of our approach, in this initial attempt, we examined whether the approach could satisfy a necessary condition, that is, if the parameters of a molecule could be reproduced based on their derived DFs. In particular, we examined whether the TIP3P water model^23^ could be reproduced using our optimization procedure starting from the parameters of the SPC/E water model. First, we derived the RDFs and EDFs of the target molecule from MD simulations. We then fitted the candidate parameters so that they could reproduce the target DFs to the greatest extent. The difference between the target DFs and those derived from the candidate parameters was minimized using a black‐box (derivative free) optimizer, that is, the covariance matrix adaptation evolution strategy (CMA‐ES).^24^, ^25^ We needed an optimization procedure that could distinguish DFs derived from similar potentials such as those obtained from the SPC/E and SPC/E_b_ water models. The detailed procedures and accuracy comparison of the RDF‐ and EDF‐based optimizations are discussed below.

## Methods

### Definition of distribution functions

We used pair DFs of the target molecule in liquid phase for parameter determination. The DFs were represented in the radial axis as the RDFs or in the energy axis as the EDFs.

RDF *ρ*(*r*) of molecules around the molecule *i* is defined as

$$\rho \left(r\right)=\frac{1}{4\pi r^{2}\rho_{0}}〈\sum_{j}\delta \left(r_{\mathrm{ij}}-r\right)〉,$$

where *ρ*_0_ is the bulk density calculated from the total number of molecules in the simulation box, 〈∙〉 represents ensemble average of the value, and *r*_*ij*_ is the distance between the center of mass of molecules *i* and *j*.

For EDF *ρ*(*e*), we employed the following definition^22^:

$$\rho \left(e\right)=〈\sum_{j}\delta \left(u\left(r_{\mathrm{ij}}\right)-e\right)〉,$$

where *u* is the interaction potential energy of molecules *i* and *j* calculated from their relative coordinates, **r**_*ij*_. According to the definition, the EDF is a distribution of pairwise energy of molecules.

For numerical calculation, each DF was defined as a histogram. Possible molecular pairs, *i* and *j*, were taken into account.

### General optimization procedure

To assess the performance of the RDF‐ and EDF‐based fittings of parameters of a molecule of interest, we employed the TIP3P water model^23^ as an example and generated the RDF and EDF as targets of fitting. Starting from parameters peripheral to those of the SPC/E water model,^7^ we assessed the fitted parameters by comparing them with the target parameters, that is, those of the TIP3P model. We fitted the vdW parameters of the oxygen atom (*σ*, *ε*), fixed charge of the oxygen atom (*q*), geometric parameters of the distance between oxygen and hydrogen atoms (*d*), and the angle between the two OH bonds (*a*). vdW parameters of hydrogen atom were set to zero because they are zero in both the TIP3P and SPC/E models. The fixed charge of hydrogen atoms was defined as −*q*/2. The parameters (*σ* [nm], *ε* [kJ/mol], *q* [*e*], *d* [nm], and *a* [degree]) of TIP3P and SPC/E are (0.315061, 0.636386, −0.834, 0.09572, and 104.52) and (0.316557, 0.650629, −0.8476, 0.1, and 109.47), respectively. We also attempted fitting the three parameters (*σ*, *ε*, and *q*) by fixing the geometry of the water molecule to that of TIP3P.

To generate the RDF and EDF of the TIP3P water model, we built a cubic box containing 1000 molecules with a density of 0.9971 g/cm^3^. We conducted three individual 100‐ns equilibrated molecular dynamics (MD) simulations, that is, Target‐Run1, Target‐Run2, and Target‐Run3, in the canonical ensemble by using the periodic boundary condition; from each of these, 10 RDFs and EDFs were generated by dividing each trajectory at an interval of 10 ns. RDFs and EDFs were generated using a conventional method with a domain of 1.5 nm and ERMOD 0.3.5,^26^ respectively. Detailed information regarding the simulation is described in the *Simulation settings* section.

The target RDF or EDF was defined as the average of the 10 RDFs or EDFs. The uncertainty of the target RDF or EDF as a function of the distance axis or energy axis was defined as the standard deviation of 10 RDFs or 10 EDFs, respectively. In summary, we obtained three target RDFs and three target EDFs with uncertainties for Target‐Run1 to Target‐Run3.

**Figure 1:**
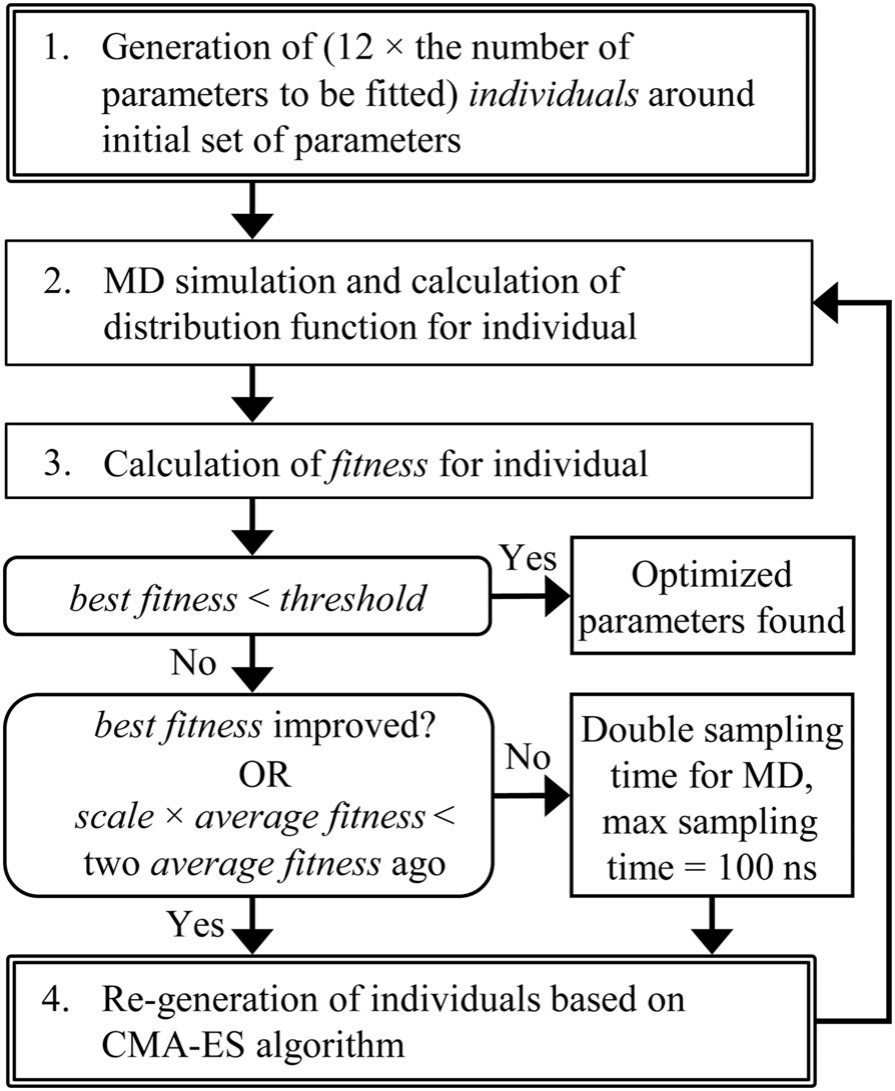
Optimization process of RDF‐ and EDF‐based derivations of force‐field parameters. See the main text for definitions of *individual*, *fitness* (eq. (1) or eq. (2)), *threshold*, *precision*, and *scale*. The *best* and *average fitness* values are the lowest and average of fitnesses in a generation, respectively. When the RDF‐based derivation is adopted, the target distribution function and the threshold regarding the RDF are employed. This holds true for the EDF‐based method. The CMA‐ES module is represented by the double‐lined boxes, that is steps 1 and 4. This module generates individuals and receives the corresponding fitness values. The number of parameters to be fitted was five for fitting of *σ*, *ε*, *q*, *d*, and *a* and three for the fitting of *σ*, *ε*, and *q* in step 1. Figure S1 of the supporting information illustrates this optimization process.

Parameter optimization was conducted as explained in Figure 1 by using an evolution strategy, wherein the candidate parameters were generated and evaluated by comparing the corresponding DF to the target DF. The generation and evaluation iterations were continued until a convergence criterion was met.

### CMA‐ES and parameter fitting

The vdW parameters (*σ* [nm], *ε* [kJ/mol]), fixed charge (*q* [*e*]), and geometric parameters (*d* [nm], *a*/500 [degree/500]) were optimized as shown in Figure 1. Here, *a* was scaled by a factor of 500 because the initial and target parameters of *a* are rather larger than those of other parameters. In the optimization process, the CMA‐ES,^24^, ^25^ a black‐box (derivative‐free) optimization algorithm that exploits an evolution strategy was employed as an optimizer. Here, we briefly explain the procedure; more detailed information on the CMA‐ES can be found elsewhere.^24^, ^25^ In each optimization step, it generates a designated number of parameter sets, called *population,* wherein each set is called an *individual*. Then, the objective function to be minimized is evaluated for the individual, and it is called *fitness*. Then, a new population is generated based on the ranking of the fitness values of previous generations. We adopted the CMA‐ES of the combined version of the rank‐*μ*‐update and rank‐one‐update algorithms^24^, ^25^ implemented in the evolutionary computation framework, DEAP 1.2.2.^27^ This combined version exploits information from *μ* individuals ranked based on fitness of the previous generation (rank‐*μ*‐update) and information from rank‐one individuals of previous generations (rank‐one‐update). Here, the ranking is relevant and quantitative information or units of fitness are disregarded. Because the search space of each population is updated according to fitness, less information is required on the domain of parameters and the responses of fitness for a slight perturbation added to different parameters (See Fig. S1 of the Supporting Information, in which the search space is adapted). When conducting the CMA‐ES procedure, we used the default settings of all parameters in DEAP 1.2.2, except for the number of individuals, which was set to 12 × the number of parameters to be fitted, and the initial covariance matrix for generating the first individuals around the parameters of the SPC/E model, the initial standard deviation for all parameters was set to 0.01 estimated from the differences between TIP3P and SPC/E, that is, 0.001496 nm for *σ*, 0.014243 kJ/mol for *ε*, 0.0136 *e* for *q*, 0.00428 nm for *d*, and 0.0099 for the scaled *a*. We considered that the initial standard deviation was applicable to the search domains of the parameter to be fitted. Although identical standard deviation was used in this study, different standard deviations can be used in the DEAP framework.

The fitness of the individuals used was defined as follows:

(1) $$\text{fitness}=\sum_{i}\frac{{\left(\rho_{i}^{\text{cand}}-\rho_{i}^{\text{target}}\right)}^{2}}{\sigma_{i}^{\text{cand}}\;\sigma_{i}^{\text{target}}}\Delta x_{i},$$

where $\rho_{i}^{\text{cand}}$ and $\rho_{i}^{\text{target}}$ are the DFs derived from candidate and target parameters as a function of bin index *i*, for which the bin size is Δx_*i*_. $\sigma_{i}^{\text{cand}}$ and $\sigma_{i}^{\text{target}}$ are the uncertainties of DFs at bin index *i*. Divided by uncertainty, the fitness becomes more robust against the problem caused by the shortness of sampling, and hence the optimization process becomes more stable. $\sigma_{i}^{\text{cand}}$ was obtained by deriving standard deviation from 10 DFs derived from 10 divided trajectories. The benefits of considering the uncertainty will be discussed later.

The fitness derived from MD trajectories still has a discrepancy from the true value that would be obtained when sufficiently large sampling was possible. This discrepancy could be called as precision. Precision is not necessarily small when the individual is far from the target parameters. In other words, the simulation length for sampling is not necessarily large. This is the case especially at the beginning of the optimization process. Hence, we started with 250‐ps sampling for calculating the DF. Moreover, we doubled the sampling time per individual of a generation if the smallest fitness value, that is, *best fitness*, of the current generation was larger than the previous best fitness, and the scaling factor × the average fitness of the current generation was greater than the average fitness of two generations ago. If the sampling time per individual of a generation exceeded 100 ns, the sampling time was reset to 100 ns. The scaling factor was defined as two (fitness^RDF^ or fitness^EDF^) or four (fitness^R × E^, see eq. 2). The optimization was iterated until the best fitness decreased more than the threshold value. The threshold was determined by choosing the largest value among fitnesses between the three target DFs described in the *General optimization procedure* section. The threshold value for the RDF‐ and EDF‐based optimization processes was 0.38, selected from (0.36, 0.38, 0.38), and 5.19 selected from (4.31, 4.87, 5.19), respectively. These values were calculated by combining Target‐Run1 and Target‐Run2, Target‐Run1 and Target‐Run3, and Target‐Run2 and Target‐Run3.

In the optimization process, satisfying the threshold value for the RDF‐based approach does not directly satisfy the threshold value for the EDF‐based approach, and vice versa because fitness^RDF^ and fitness^EDF^ for a slight added perturbation respond differently. To obtain fitted parameters that almost satisfy both thresholds, we introduced the following definition of fitness:

(2) $$\text{fitnes}s^{R\times E}=\text{fitnes}s^{\mathrm{RDF}}\;\text{fitnes}s^{\mathrm{EDF}},$$

where the fitnesses calculated using RDF and EDF using eq. (1) are multiplied. Optimization was conducted until the multiplied value of both thresholds was satisfied.

### Simulation settings

The initial cubic box containing 1000 water molecules was structurally minimized using the steepest descent algorithm. The minimized system was subjected to a three‐step equilibration, that is, 50‐ps MD simulation with a time step of 0.5 fs, applying random initial velocity that gave the Maxwell–Boltzmann distribution at 298 K, 50‐ps MD simulation with a time step of 1 fs, and 200‐ps MD simulation with a time step of 2 fs. Small time steps were used to relax the system that was unstable sometimes owing to the parameters assigned by the CMA‐ES module. The equilibrated system was subjected to a production run with a time step of 2 fs with a snapshot structure recorded for analysis at every 0.5 ps. The simulation length of the production run was set, as explained in the previous section, or was set to 100 ns, when the target RDFs and EDFs were generated.

During the simulation, the vdW interaction was smoothly switched to zero starting from 0.9 to 1.0 nm. The electrostatic interaction was calculated using the particle mesh Ewald^28^, ^29^ with a real space cutoff of 1 nm, reciprocal space grids of 20 for *x*, *y*, and *z* directions, an interpolation order of 4, and a Gaussian width of 0.320163 nm. The equation of motion was integrated using the leap‐frog algorithm^30^ by controlling the temperature at 298 K by using the velocity rescaling algorithm^31^ with a coupling time of 0.1 ps. The distances between the oxygen and hydrogen atoms and between the two hydrogen atoms were fixed using the settle algorithm.^32^ All minimizations and simulations were conducted using GROMACS 2018.1^33^ and were terminated normally.

## Results and Discussion

### Comparison of fitted parameters and convergence

**Figure 2:**
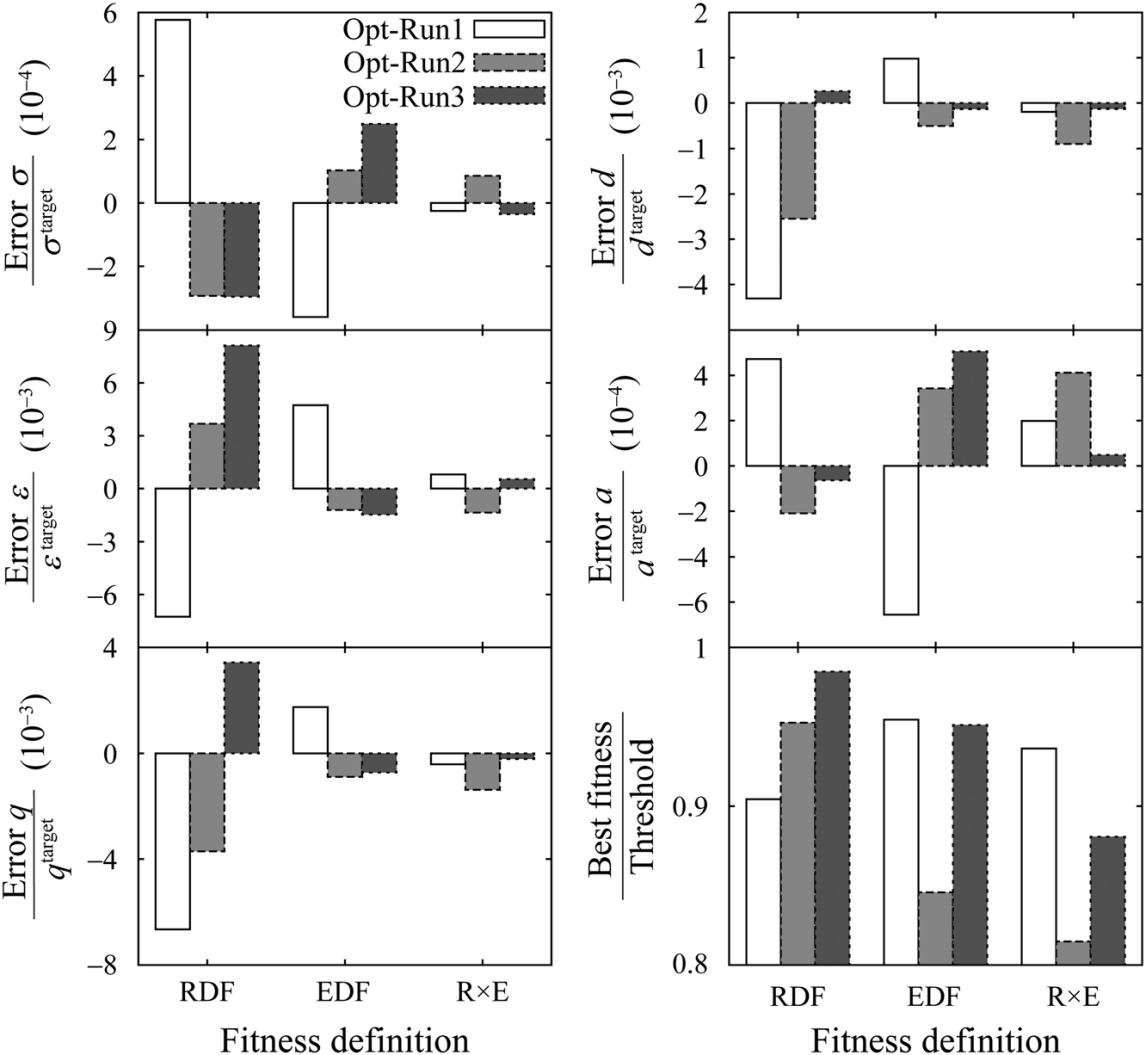
Error ratios of fitted parameters, *σ*, *ε*, *q*, *d*, and *a*, and the corresponding target parameters. The error is defined as (fitted parameter—target parameter). The plots also show the fitness relative to the threshold of each fitness defined when these parameters are determined.

**Figure 3:**
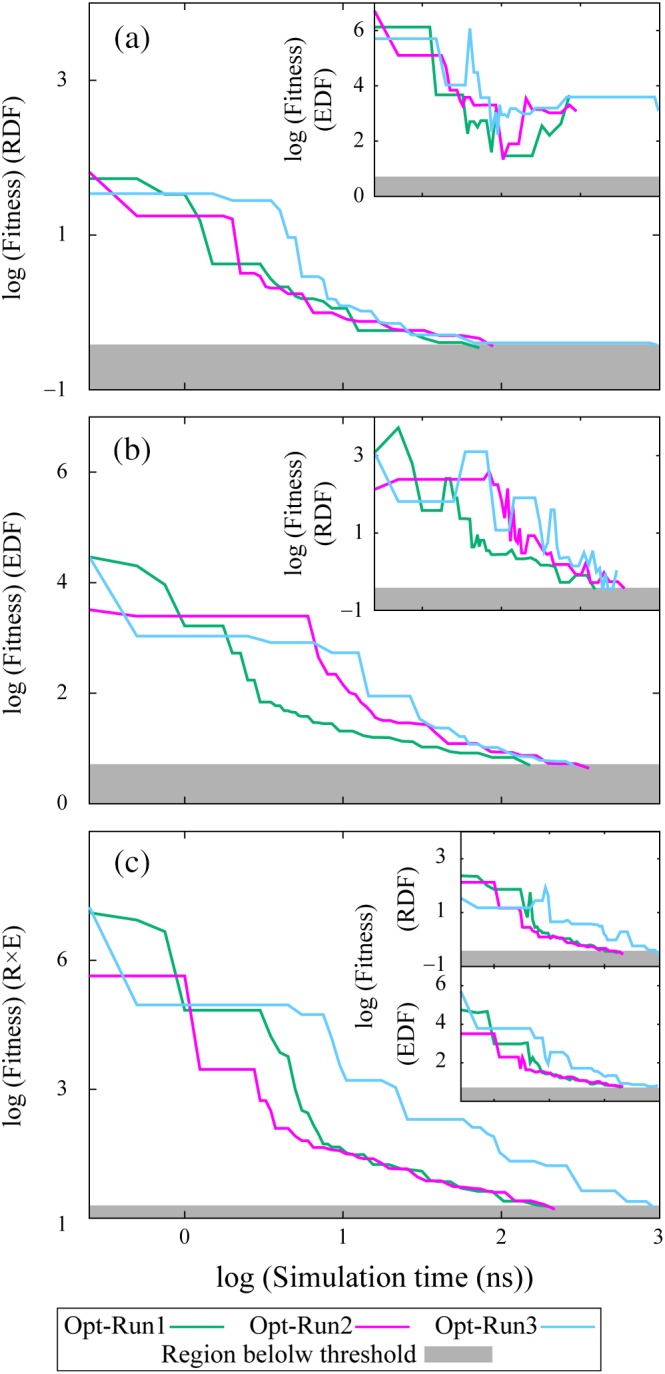
Convergence of fitness as a function of cumulative simulation time over the generations per individual. Here, fitted parameters are *σ*, *ε*, *q*, *d*, and *a*. The optimization used the (a) RDF‐, (b) EDF‐, and (c) R × E‐based fitnesses. In the insets, the (a) EDF‐, (b) RDF‐, and (c) RDF‐ and EDF‐based fitnesses are shown. The cumulative simulation times are tabulated in Table S2 of the Supporting Information.

We conducted individual optimization three times (hereinafter referred to as Opt‐Run1 to Opt‐Run3). Opt‐Run1 to Opt‐Run3 employed target DFs derived from Target‐Run1 to Target‐Run3, respectively. After iterating generations and evaluations of optimization processes several times, as described in Figure 1, the fitness value for each fitness definition reached the corresponding threshold. The comparison of the RDF‐ and EDF‐based fitnesses showed that the EDF provided more accurate parameters for *σ*, *ε*, *q*, and *d*, but not for *a*, for which the more accurate value was obtained using the RDF‐based definition, as shown in Figure 2. We found that the convergence of the RDF‐based fitness did not lead to the convergence of the EDF‐based fitness, as shown in Figure 3a and its inset. On the other hand, convergence of the EDF‐based fitness to its threshold value almost made the RDF‐based fitness converge to its threshold value. Hence, we concluded that the EDF‐based fitness provides more accurate parameters than the RDF‐based fitness.

We observed a small deviation from the threshold at the final steps of the RDF‐based fitness for the EDF‐based optimization (the inset of Fig. 3b). The deviation was quenched using the R × E‐based fitness (insets of Fig. 3c). The convergence of the R × E‐based fitness resulted in a smoother decrease of the RDF‐ and EDF‐based fitnesses. The accuracy of the obtained parameters was improved or at least did not worsen more than those obtained through the RDF‐ or EDF‐based fitnesses. Hence, among three fitness definitions, the R × E outperformed the others on an average. The parameters obtained through the R × E‐based fitness approached the target parameters well. The maximum ratios of errors obtained among the three individual optimizations were 0.9 × 10^−4^, −1.4 × 10^−3^, −1.4 × 10^−3^, 0.9 × 10^−3^, and 4 × 10^−4^, which were at least 10‐fold smaller than the ratios of error for the parameters of SPC/E (initial parameters), that is, 47 × 10^−4^, 22 × 10^−3^, 16 × 10^−3^, −43 × 10^−3^, and 47 × 10^−4^ for *σ*, *ε*, *q*, *d*, and *a*, respectively. These ultimately obtained error ratios showed that the optimization procedure can distinguish the DFs of highly similar water models such as SPC/E and SPC/E_b_, the difference between which is a 1% increase in the OH bond length.^8^

We observed the correlation that when an error of a parameter derived using a certain target DF was relatively larger, an error of another parameter derived using the same target DF also increased. The most correlated errors were those of *σ* and *ε* with a correlation coefficient of −0.94, which was calculated using the nine errors described in Figure 2 for *σ* and *ε*. The second‐most correlated errors were *q* and *d* with a correlation coefficient of 0.93. These correlations were not related to the final fitness compared to the corresponding threshold, as shown in Figure 2. The correlation would occur because some parameters correlate with each other, and the effect of correlation would be eliminated by fixing one of the correlated parameters. We conducted optimization only for *σ*, *ε*, and *q* by fixing *d* and *a* to those of TIP3P by using the same three target DFs. We found a small improvement in the accuracy of the fitted *σ* and *ε* compared with those obtained when all five parameters were subjected to the optimization, as shown in Figure S2 of the Supporting Information. On the other hand, the accuracy of *q* improved by one to two orders of magnitudes. This suggests that excluding the correlated parameters from the fitting would be one option to obtain accurate parameters. In this case, the EDF‐based optimization was slightly better than the R × E‐based optimization.

### Comparison of properties derived with fitted parameters

To evaluate how errors of fitted parameters affect the representative properties of the target molecule, we derived the molecular dipole moment, pressure, and solvation free energy (SFE) of a single molecule using the fitted parameters. The pressure was derived from the simulation corresponding to the fitted parameter during optimization. The SFE was calculated using the free energy perturbation combined with the multistate Bennett acceptance ratio method^34^ implemented in pymbar 3.0.3^34^ and Alchemical Analysis 1.0.2,^35^ the procedure and obtained SFEs for which are detailed in Table S1 of the Supporting Information.

**Figure 4:**
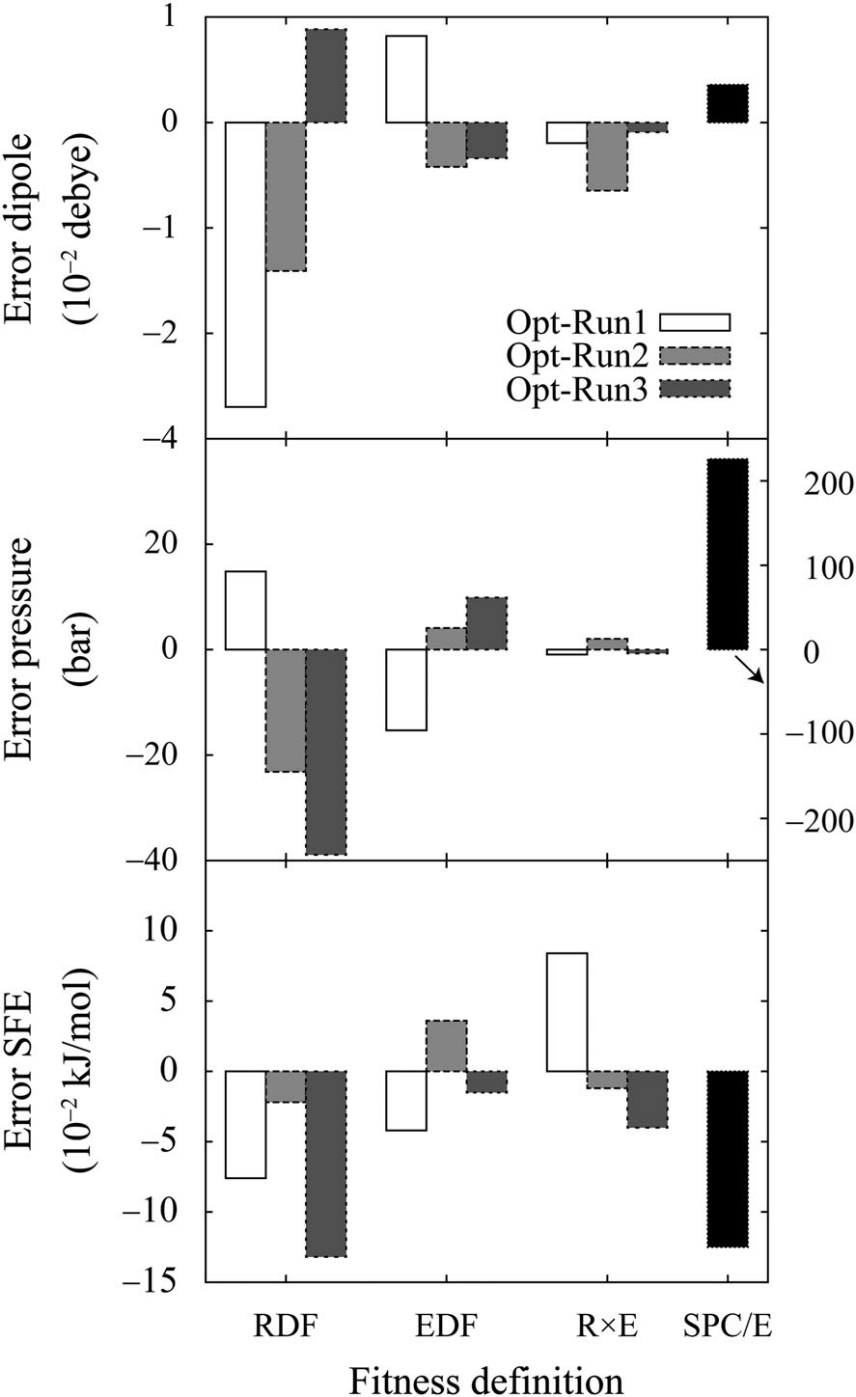
Errors of properties derived using fitted parameters. (a) Errors of the molecular dipole moment; (b) pressure obtained when the canonical ensemble simulation was conducted; and (c) solvation free energy (SFE). The error is defined as (property derived from fitted parameters—property derived from the target parameters).

The errors observed for parameters derived with the RDF‐based fitness were larger than those observed for the EDF‐based and R × E‐based fitnesses, as shown in Figure 4. This observation was consistent with the relative magnitude of errors in parameters, as shown in Figure 2. The errors in the molecular dipole moment and SFE, and in the EDF‐based and R × E‐based optimizations were comparable. On the other hand, the errors in the pressure derived using the R × E‐based optimization were smaller than those with the EDF‐based optimization. It is difficult to relate the errors in these properties and the parameters used. However, a clear positive correlation between the errors in pressure and the errors in *σ* were observed. We also individually derived these properties using the parameters obtained through the simultaneous optimizations for *σ*, *ε*, and *q*. As a result, the EDF‐based optimization outperformed the others as shown in Figure S4 of the Supporting Information. In both the optimization for five and three parameters, we concluded that considering the information of EDF is essential for the accurate determination of parameters.

### Bin definitions and fitness of target parameters

We found that the fitness calculation depends on the bin definition of DF, for example, with smaller bin sizes, the DF can capture the details of true distributions, whereas the uncertainty corresponding to each bin increases. We studied 11 types of definitions for RDF, that is, bin size = (1, 2, 4, 5, 10, 20, 40, 50, 100, 200, 500) × 10^−5^ nm. The RDF was defined from 0 to 1.5 nm. For the EDF, 200 types of definitions were studied, as listed in Table 1. In the previous sections, the employed bin definitions were selected based on the consideration mentioned in the subsequent sections.

**Table 1:** Bin definition of energy‐distribution‐function searched for analysis of bin dependency of fitness.

Region (kcal/mol)	−30.02 to −0.22	−0.22 to −0.02	−0.02 to 0.02	0.02 to 0.22	0.22 to 30.22
Minimum bin size (kcal/mol)	0.04	0.002	0.0002	0.002	0.04
Bin size (kcal/mol)	0.04	0.002 × *I*_*i*_	0.0002 × *J*_*j*_	0.002 × *K*_*k*_	0.04
Variation of bin definition		*i* = {1, …, 5} *I*_*i*_ = {1, 2, 4, 10, 20}	*j* = {1, …, 8} *J*_*j*_ = {1, 2, 4, 8, 20, 40, 100, 200}	*k* = {1, …, 5} *K*_*k*_ = {1, 2, 4, 10, 20}	

Bin definition ID = 5 × 8 × (*i* − 1) + 5 × (*j* − 1) + *k*. Note that only in this table, unit for energy is kcal/mol (1 cal = 4.184 J).

**Figure 5:**
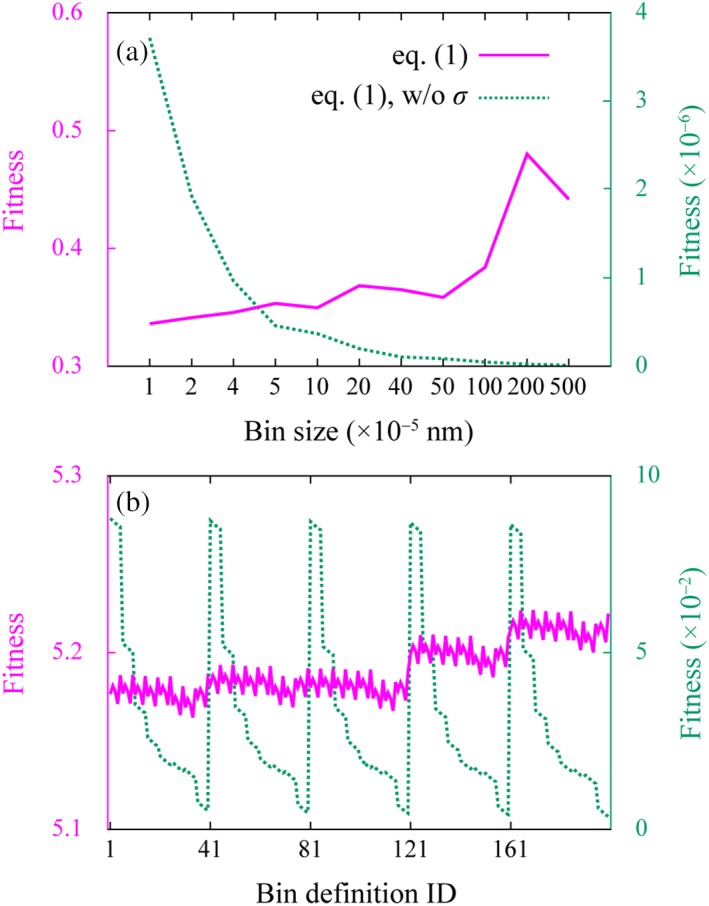
Fitness defined as eq. (1) of the target molecule, calculated using various bin definitions, is plotted for the (a) RDF‐ and (b) EDF‐based approaches. In addition, fitness calculated without the uncertainty of distribution function (eq. (1), w/o *σ*) is plotted. The fitness was defined as the maximum fitness among three pairs of three individual simulations, that is, Target‐Run1 and Target‐Run2, Target‐Run1 and Target‐Run3, and Target‐Run2 and Target‐Run3.

We conducted three individual 100‐ns simulations of the TIP3P water box, Target‐Run1 to Target‐Run3, and calculated the fitnesses of three pairs from among them by using eq. (1). Their maximum value was defined as representative of the fitness for its bin definition. We observed a trivial dependency of fitness on bin definitions, as shown in Figure 5. Smaller bin sizes gave slightly smaller (better) fitness. The ratio between the maximum and minimum RDF‐based fitnesses calculated using various bin definitions was 1.43. The corresponding value of the EDF‐based fitness was 1.01. The RDF‐based fitnesses calculated using bin sizes of 0.02 and 0.05 nm were significantly larger than those calculated using smaller sizes. This could reflect the loss of ability to capture the details of the true DF. Hence, bin sizes between 0.00001 and 0.01 nm were selected for the RDF‐based fitness.

Interestingly, when uncertainty in eq. (1) was not considered, that is, when $\sigma_{i}^{\text{cand}}=\sigma_{i}^{\text{target}}=1$, the dependency was relatively different. Better fitnesses could be obtained with larger bin sizes. The ratio between the maximum and minimum RDF‐ and EDF‐based fitnesses was 361 and 23.5. This is simply because larger bin sizes achieved statistically more stable values, and ${\left(\rho_{i}^{\text{cand}}-\rho_{i}^{\text{target}}\right)}^{2}$ decreased. Without uncertainty, the details of the true DF would be buried in this statistical effect. The results show that without uncertainty, the robustness of fitness for bin definitions can deteriorate. Hence, we decided to consider uncertainty, as in eq. (1). Note that the percentage of EDF‐based fitness values contributed from the regions not involved in the analysis of bin definition, that is (−30.02) to (−0.22) and 0.22 to 30.22, was approximately 97%–98% when considering uncertainty. This indicates that there would be a room for fine‐tuning of bin‐size definition in future research.

### Bin definitions and response of fitness for perturbation on parameters

We evaluated the response of fitness when a perturbation was added to a single parameter. We conducted a 100‐ns simulation using parameters including the perturbed one and evaluated fitness between the derived DF and three target DFs derived beforehand as functions of various bin definitions. Their minimum value was defined as the representative of fitness for its bin definition. The perturbations that we employed were ±0.01% for *σ*, *q*, *d*, and *a*, and ±0.05% for *ε*. As the response of a perturbation of 0.01% for *ε* was too small both for the RDF‐ and EDF‐based approaches, we used the fivefold larger perturbation for *ε*. In addition, the response of fitness was evaluated when the SPC/E parameters were used.

**Figure 6:**
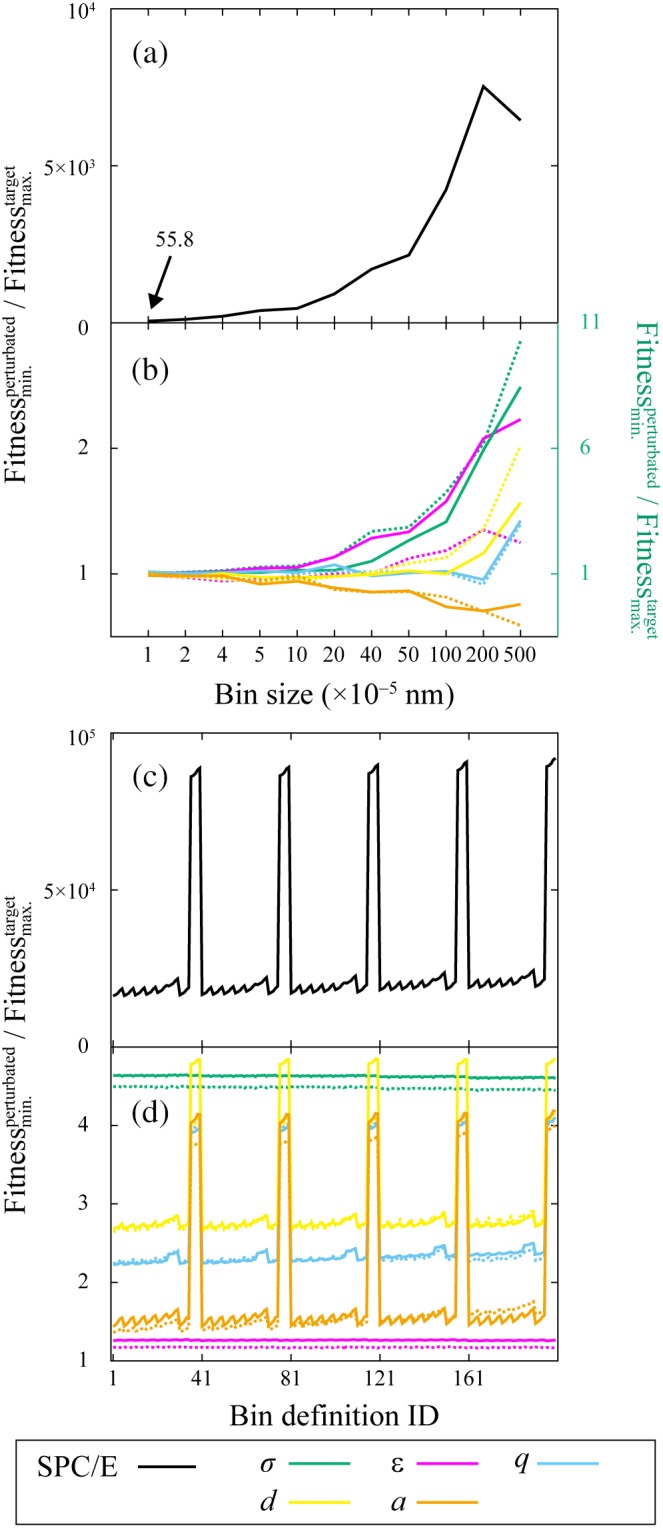
Ratio of the fitness of perturbed parameters added to the target parameters and the fitness between target parameters of individual simulations are plotted for (a, b) RDF‐based and (c, d) EDF‐based optimizations as functions of various bin definitions. Three target distribution functions were derived from three individual runs (Target‐Run1–Target‐Run3), respectively, and therefore, we had three fitnesses of the perturbed parameters, among which the minimum was used for the ratio calculation. The maximum among the fitnesses between target DFs was used for the ratio calculation. The perturbation for *σ*, *q*, *d*, and *a* is ±0.01% and that for *ε* is ±0.05%. The positively and negatively added perturbations correspond to solid and dashed lines, respectively.

The comparison of the fitnesses of the SPC/E parameters and target parameters showed that any bin definition for both the RDF‐ and EDF‐based optimizations could distinguish them, as shown in Figures 6a and 6c. For the RDF‐based approach, we observed the tendency of increased distinguishability for these parameters when the bin size was increased. For the EDF‐based approach, the distinguishability improved remarkably when the region of −0.02 to 0.02 was treated as a single bin with a bin size of 0.04, which corresponded to *j* = 8 (*J*_8_ = 200) in Table 1. This is because the uncertainty of the bins around 0 had remarkably decreased, for example, the average of values and standard deviations in this region for bin sizes of 0.0002 (*J*_1_ = 1), 0.02 (*J*_7_ = 100), and 0.04 (*J*_8_ = 200) were 3.11 ± 0.0006, 311 ± 0.026, and 622 ± 0.005, respectively. This made the value of $\Delta x_{i}/\left(\sigma_{i}^{\text{cand}}\;\sigma_{i}^{\text{target}}\right)$ in eq. (1) larger for the bin size of 0.04.

In the case that a small perturbation was added to each parameter, the RDF‐based approach showed dependency on the bin definitions, as shown in Figure 6b. We observed a similar tendency of greater responses of *σ*, *ε*, and *d* with larger bin sizes. However, the tendency was not observed, especially for *a,* where the fitness responded oppositely. This means that bin definition should be chosen carefully. In the parameter optimization, we used the bin size of 0.01 nm. In this case, the RDF‐based approach can distinguish parameters with a small perturbation added to *σ*, *ε*, and *d* from the original. However, it cannot distinguish parameters with a small perturbation added for *q* and *a* from the original. For the EDF‐based approach, the shape of the dependency of bin definitions for *q*, *d*, and *a* was similar to that obtained when SPC/E parameters were used, as shown in Figures 6c and 6d. Interestingly, the responses observed for *σ* and *ε* were relatively flat. For any perturbed parameters, the EDF‐based approach could distinguish them from the original. We used the bin definition of *i* = 1, *j* = 8, and *k* = 5 (the bin definition ID = 40) in Table 1. This definition resulted in relatively smaller fitness values between the DFs derived among individual simulations of the target parameters (Fig. 5b) and a larger response when a small perturbation was added (Fig. 6d).

According to the results in Figures 6b and 6d, the EDF‐based approach could achieve more accurate parameters. The results correlate with the actual accuracies of the RDF‐ and EDF‐based approaches obtained through the optimization process, as shown in the previous sections.

## Conclusions

We confirmed that a set of force‐field parameters for a target molecule can be determined by adjusting candidate parameters such that they reproduce the target DF of the pair of molecules, taking the water molecule as an example target. Conventionally, the DF represented in the radial axis (RDF) was used for such purposes. In this study, we showed the benefits of introducing the DF represented in the energy axis (EDF).

We fitted parameters of energy functions targeting the RDF and/or EDF derived for the TIP3P water model starting from the parameters of the SPC/E water model, employing a black‐box (derivative‐free) optimizer, the CMA‐ES, to search for candidate parameters that can reproduce the target DFs. As a result, more accurate parameters were yielded when the residual sum of the squares of EDFs between the candidate and target DFs was minimized, in which uncertainty in the DFs originating from the shortness of the structure sampling was considered. This incorporation of uncertainty can improve conventional RDF‐based fittings such as the iterative Boltzmann inversion (IBI). A more accurate fitting was achieved owing to the higher sensitivity of the EDF than that of the RDF for a small perturbation added to a set of the parameters of the molecule. The discovery of the benefits of introducing the EDF provides valuable insights into the class of coarse‐graining approaches—the so‐called bottom–up approaches^5^—that fit parameters using information obtained from a higher‐level calculation than that of the developed force field. However, given that this work was performed only under a limited set of conditions, that is, determination of parameters to reproduce the DFs of TIP3P water, the applicability of EDF‐based optimization should be tested in more demanding situations, such as the coarse graining of a molecule by using the target DF obtained through the corresponding fine‐grained description; this would involve the use of quantum mechanics. For example, the fragment molecular orbital (FMO) method can be used to determine the ab initio quantum mechanics‐based pairwise interaction energies of molecules (fragments) for a large system such as the one that includes more than thousands of water molecules^36^; this can be used to derive the EDF of the molecules.

The computational cost for searching the candidate parameters that reproduced the target DF is not trivial. Thus, reducing the computational cost must be investigated for practical application of the force‐field determination. The optimization requiring the longest cumulative simulation time of 963 ns per individual over the generations (Fig. 3c, Opt‐Run3) resulted from the repeated 100‐ns samplings for the last several generations. This suggests that there is a room of improvement in the optimization procedure, especially at the step where simulation time was doubled, as shown in Figure 1. Likewise, pursuing a more appropriate optimizer and a judicious minimizing algorithm other than black‐box optimization would be desirable.

## Supporting information


**Appendix S1** Supporting Information: See supplementary material for an example of the parameter optimization, cumulative time and the number of generations for convergence, the results of the simultaneous optimization for *σ*, *ε*, and *q,* and the detailed procedure of the solvation free energy calculation.
