# Supplementary material for: Force‐field parametrization based on radial and energy distribution functions
Source: J Comput Chem. 2019 Jul 25;40(29):2577–85. doi: 10.1002/jcc.26035 (PMC6790663; doi:10.1002/jcc.26035)
Supplement: Supplementary file 1 — Appendix S1 Supporting Information: See supplementary material for an example of the parameter optimization, cumulative time and the number of generations for convergence, the results of the simultaneous optimization for σ, ε, and q, and the detailed procedure of the solvation free energy calculation. [file JCC-40-2577-s001.pdf]

Supporting information

Force-field parametrization based on radial and energy distribution functions

Shuntaro Chiba,[1,2] Yasushi Okuno,[1,3] Teruki Honma,[1] Mitsunori Ikeguchi[1,4]

1. RIKEN Medical Sciences Innovation Hub Program, 1-7-22, Suehiro-cho, Tsurumi-ku, Yokohama, 230-0045, Japan
2. RIKEN Cluster for Science and Technology Hub, 6-3-5, Minatojima-minamimachi, Chuo-ku, Kobe, Hyogo, 650-0047, Japan
3. Graduate School of Medicine, Kyoto University, Shogoin-Kawaharacho, Sakyo-ku, Kyoto, 606-8507, Japan
4. Graduate School of Medical Life Science, Yokohama City University, 1-7-29, Suehiro-cho, Tsurumi-ku, Yokohama, 230-0045, Japan

Correspondence to: Shuntaro Chiba (E-mail: shuntaro.chiba@riken.jp)

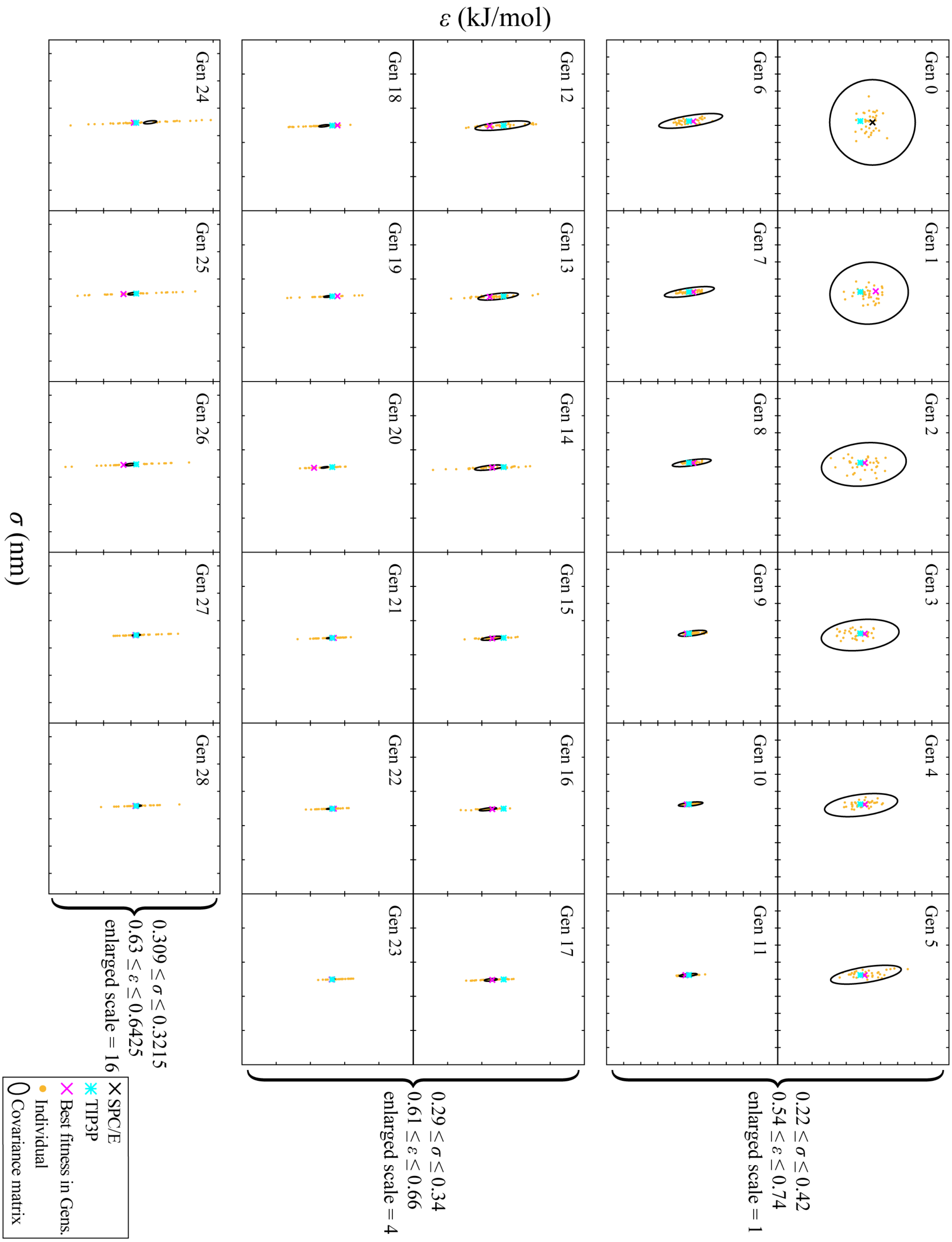

Figure S1. Optimization process of σ and ε using the CMA-ES. Parameters σ, ε, and q were fitted using the R×E fitness (Eq. 2, Opt-Run1). In Generation (Gen) 0, population was generated to reproduce the given covariance matrix, and then the fitness for each individual was calculated. In subsequent generations, a new covariance matrix was generated using the rank-μ-update and rank-one-update algorithms.[1,2] When generating a population, the overall scale of the matrix was considered, sometimes producing individuals far outside the covariance matrix.

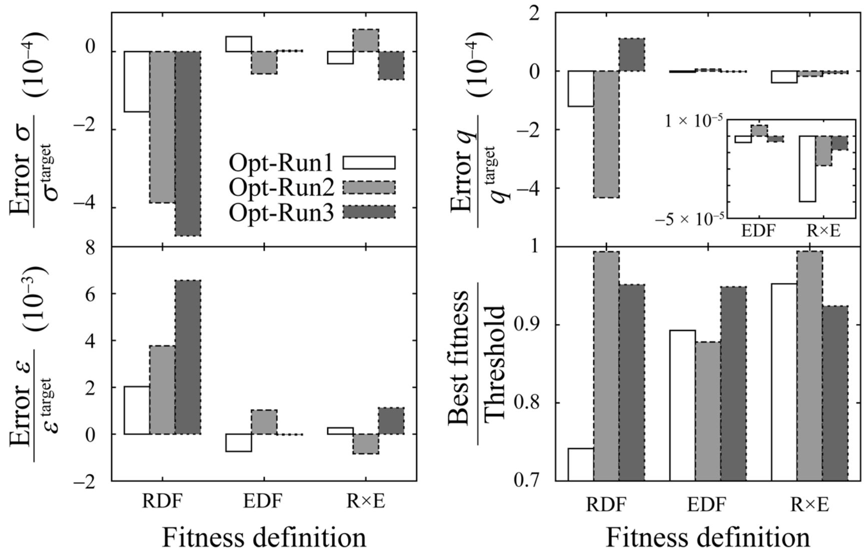

Figure S2. Ratios of the error of fitted parameters σ, ε, and q and the corresponding target parameters. The error is defined as (fitted parameter − target parameter). Fitness relative to the threshold of each fitness definition when these parameters were determined are also plotted.

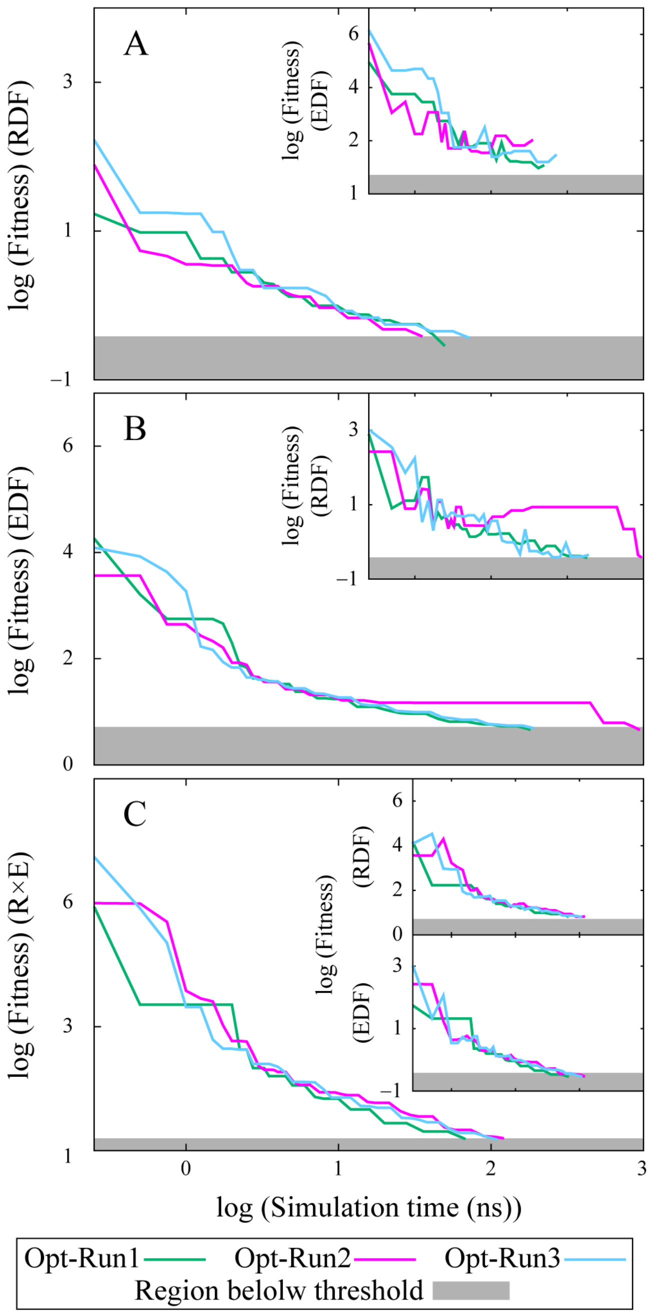

Figure S3. Convergence of fitness as a function of accumulative simulation time per individual. Here, σ, ε, and q are optimized, using the (A) RDF-based, (B) EDF-based, and (C) R×E-based fitnesses. The insets show the (A) EDF-based, (B) RDF-based, and (C) RDF- and EDF-based fitnesses.

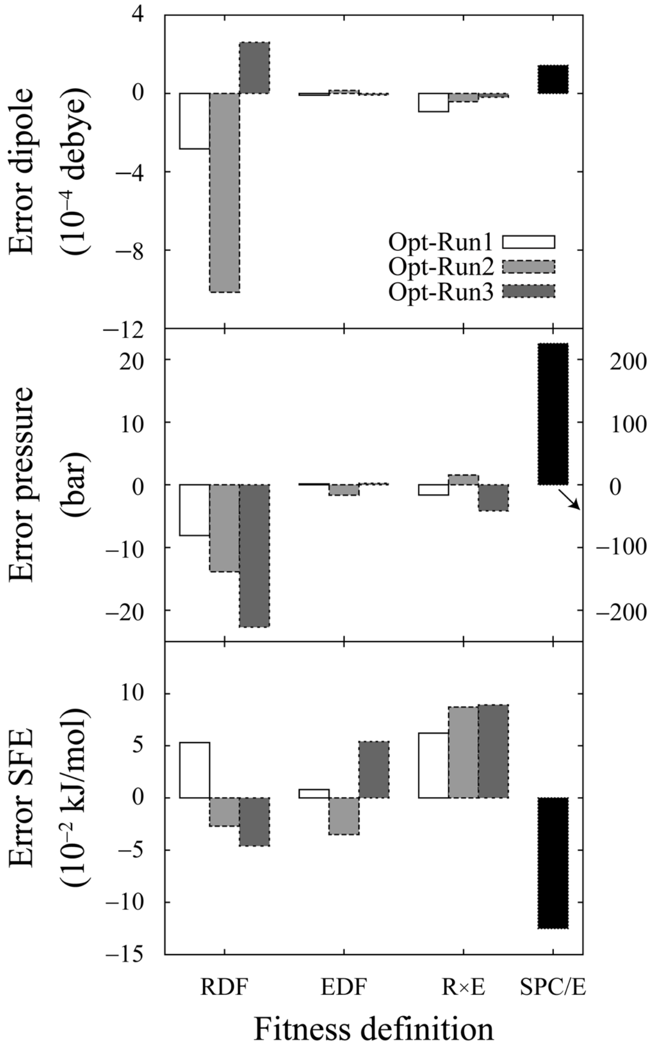

Figure S4. Errors of properties derived using the fitted parameters. Errors of the (A) molecular dipole moment, (B) pressure obtained when the canonical ensemble was simulated, and (C) solvation free energy (SFE). Error is defined as (property derived from fitted parameters − property derived from the target parameters).

Procedure for free energy perturbation

Solvation free energy (SFE) of a water molecule described with respect to fitted parameters was calculated using the free energy perturbation combined with the multistate Bennett acceptance ratio method[3] implemented in pymbar 3.0.3[3] and Alchemical analysis 1.0.2.[4] The vdW and electrostatic interactions were simultaneously switched using the following lambda values: 0, 0.111111, 0.222222, 0.333333, 0.444444, 0.555556, 0.627778, 0.7, 0.733333, 0.766667, 0.8, 0.825, 0.85, 0.875, 0.9, 0.925, 0.95, 0.9625, 0.96875, 0.975, 0.98125, 0.9875, 0.991667, 0.995833, and 1. To avoid singularity in simulations for 0 < lambda < 1, the soft-core potential implemented in GROMACS was employed. The procedure used for the equilibration and production that was run in each lambda simulation was almost the same as that described in section *Simulation settings,* with two exceptions that the length of the production run was set to 16 ns and a single water molecule defined as a solute was subjected to the LINCS constraint[5,6] to fix its geometry instead of the SETTLE constraint. This is because GROMACS does not allow use of the SETTLE for multiple molecule types. The simulation length for the target molecule, the TIP3P water, was set to 100 ns to obtain a precise value. The sampling coordinates used for the free energy calculation were automatically collected through the Alchemical analysis so that they were not correlated energetically. The above-mentioned simulation setting achieved very good overlap in phase space and small uncertainty as shown in Table S1.

Table S1. Solvation free energy calculated using free energy perturbation

Parameters fitted	Fitness definition	Run ID	Solvation free energy kJ/mol	Uncertainty kJ/mol	Smallest overlap in phase space
σ, ε, q, d, a	RDF	Opt-Run1	–25.37	0.029	0.55
		Opt-Run2	–25.31	0.028	0.53
		Opt-Run3	–25.42	0.028	0.54
	EDF	Opt-Run1	–25.33	0.028	0.54
		Opt-Run2	–25.26	0.028	0.53
		Opt-Run3	–25.31	0.029	0.53
	R×E	Opt-Run1	–25.21	0.029	0.55
		Opt-Run2	–25.30	0.028	0.54
		Opt-Run3	–25.33	0.028	0.53
σ, ε, q	RDF	Opt-Run1	–25.28	0.028	0.54
		Opt-Run2	–25.33	0.029	0.54
		Opt-Run3	–25.24	0.028	0.54
	EDF	Opt-Run1	–25.24	0.028	0.53
		Opt-Run2	–25.32	0.028	0.54
		Opt-Run3	–25.34	0.028	0.54
	R×E	Opt-Run1	–25.23	0.029	0.54
		Opt-Run2	–25.20	0.028	0.54
		Opt-Run3	–25.20	0.029	0.55
SPC/E			–25.42	0.031	0.54
TIP3P			–25.29	0.010	0.55

Definitions of uncertainty and overlap in phase space can be referred to from relevant literature.[4]

Table S2. Generations and cumulative simulation time per individual necessary for convergence

Parameters fitted	Fitness definition	Run ID	Number of generations	Cumulative simulation time per individual (ns)
σ, ε, q, d, a	RDF	Opt-Run1	30	72.5
		Opt-Run2	29	88.5
		Opt-Run3	43	963
	EDF	Opt-Run1	42	151.5
		Opt-Run2	39	358
		Opt-Run3	39	286.5
	R×E	Opt-Run1	39	199.5
		Opt-Run2	47	217.5
		Opt-Run3	41	921.5
σ, ε, q	RDF	Opt-Run1	27	49.75
		Opt-Run2	26	35.5
		Opt-Run3	25	72.25
	EDF	Opt-Run1	38	181.25
		Opt-Run2	35	946.5
		Opt-Run3	39	192
	R×E	Opt-Run1	29	68
		Opt-Run2	39	121.5
		Opt-Run3	36	110

References

1. Hansen, N.; Ostermeier, A. *Evol. Comput.* **2001**, 9(2), 159-195.
2. Hansen, N. *ArXiv e-prints* **2016**, arXiv:1604.00772, 02016.02005.
3. Shirts, M. R.; Chodera, J. D. *J. Chem. Phys.* **2008**, 129(12), 124105.
4. Klimovich, P. V.; Shirts, M. R.; Mobley, D. L. *J. Comput. Aided. Mol. Des.* **2015**, 29(5), 397-411.
5. Hess, B.; Bekker, H.; Berendsen, H. J. C.; Fraaije, J. G. E. M. *J. Comput. Chem.* **1997**, 18(12), 1463-1472.
6. Hess, B. *J. Chem. Theory Comput.* **2008**, 4(1), 116-122.
